# Knowledge, Attitudes, and Clinical Practices Regarding Food–Drug, Herb–Drug, and Supplement–Drug Interactions: A Pilot Cross-Sectional Study Among Healthcare Professionals in Portugal

**DOI:** 10.3390/nu18162600

**Published:** 2026-08-08

**Authors:** Walter Silva, Maria João Campos, Angelina Pena

**Affiliations:** 1 Faculty of Pharmacy, Azinhaga de Santa Comba, Health Sciences Campus, University of Coimbra, 3000-548 Coimbra, Portugal; waltermsilva@ulscoimbra.min-saude.pt (W.S.); apena@ff.uc.pt (A.P.); 2 Hospitais da Universidade de Coimbra—Unidade Local de Saúde de Coimbra, Praceta Prof. Mota Pinto, 3004-561 Coimbra, Portugal; 3 LAQV-REQUIMTE, Laboratory of Bromatology, Pharmacognosy and Analytical Sciences, Faculty of Pharmacy, University of Coimbra, 3000-295 Coimbra, Portugal

**Keywords:** drug interactions, food–drug interactions, herb–drug interactions, supplement–drug interactions, food supplements, healthcare professionals, patient safety

## Abstract

**Background/Objectives**: The concomitant use of conventional medicines with foods, food supplements, and herbal products has increased, raising concerns about interactions. Despite this, healthcare professionals’ knowledge and clinical practices in this domain remain incompletely characterised. This study aimed to assess knowledge, attitudes, and clinical practices regarding food–drug, herb–drug, and supplement–drug interactions among healthcare professionals (HPs) in Portugal. **Methods**: A cross-sectional pilot study was conducted among 311 HPs using an online questionnaire; participants were recruited through convenience and snowball sampling. The instrument evaluated perceptions, practices, and knowledge (22 items; score 0–22). Descriptive statistics and non-parametric tests were used for group comparisons. **Results**: Most participants (90.6%) recognised the clinical relevance of herb–drug interactions; however, only 57.6% reported routinely asking patients about food supplements or herbal product use, and 46.0% about teas or infusions. The median knowledge score was 9.00 (interquartile range 9.00), with a mean of 9.34 ± 5.91. Significant differences were observed between professions (*p* < 0.001): pharmacists achieved the highest scores and nurses the lowest. In exploratory analyses, academic training and continuing education were associated with higher knowledge, whereas professional experience was not. **Conclusions**: Among the professionals studied, a discrepancy exists between perceived clinical relevance and actual practice, with important gaps in technical knowledge. Within the limits of this sample, the findings point to a need for improved education and structured clinical approaches to enhance patient safety when medicines and natural products are used concomitantly.

## 1. Introduction

Food supplements (FS) are regulated in the European Union (EU) as foodstuffs intended to supplement a normal diet and provide concentrated sources of nutrients or other substances with a nutritional or physiological effect, marketed in dose form [1]. Their consumption has increased consistently over recent decades in many countries [2], including Portugal [3,4], driven by easier access to information, e-commerce, marketing, and the growing influence of social media [5,6,7].

In Portugal, the National Food and Physical Activity Survey (IAN-AF 2015–2016) reported that 26.6% of the population had consumed FS in the previous 12 months [8]. Internationally, a 2022 survey across 14 EU member states (excluding Portugal) reported that approximately 88% of respondents had used an FS at least once in their lifetime [9].

This pattern is consistent with international data, including recent United States (US) data showing rising supplement use and polysupplementation among adults [10]. Common motivations include health promotion, disease prevention, and the optimisation of physical or cognitive performance [11,12].

The growing use of FS within self-care practices, particularly when combined with conventional medicines, chronic disease, or polypharmacy, raises clinically relevant concerns regarding potential supplement–drug interactions [4]. Although FS are legally classified as food products, their bioactive ingredients, dose form presentation, claimed physiological effects, and frequent association with nutrition-, health-, and disease-risk-related claims may blur, from the consumer’s perspective, the distinction between ordinary foods and health-promoting products [13]. This issue is compounded by the fact that FS and herbal products are frequently under-reported by patients and undervalued in clinical practice. A substantial proportion of users do not disclose FS or herbal product use to healthcare professionals (HPs), with non-disclosure rates reported to reach 40–70%, often because they are not asked, perceive these products as safe, or do not consider them clinically relevant [14].

From a regulatory perspective, four categories can be distinguished: ordinary foods; food supplements; herbal preparations marketed as herbal food supplements under food law [1]; and herbal medicinal products, which fall under medicines legislation and require registration or marketing authorisation [15]. Importantly, these categories are not determined by the botanical or chemical identity of the constituent alone; the same plant or substance may be presented as a food, a food supplement, or a herbal medicinal product depending on its formulation, dose, and accompanying claims, so that its regulatory classification and the applicable evidentiary standard follow from presentation and intended function. This distinction is not merely administrative: because herbal food supplements are regulated as foods and herbal medicines as medicinal products, the evidence base and product information available to support their safe use differ substantially [16].

Herbal medicines are widely used because of their acceptance as complementary or alternative therapeutic options [17]. However, unlike conventional medicines, the authorisation of which depends on robust demonstration of quality, safety, and efficacy, a significant proportion of herbal medicines, particularly those classified under the traditional use framework, do not have comparable clinical evidence of efficacy and safety [15,18]. Consequently, herb–drug interactions (HDIs) may result in clinically relevant adverse outcomes, including adverse effects, toxicity or reduced therapeutic response; however, the assessment of causality in this context remains challenging [19].

The use of FS and herbal products is of paramount importance during the preoperative period, and medical and nursing staff should possess a comprehensive understanding of potential complications and side effects to prevent them. Certain herbal medicines with pharmacological activity are addressed in preoperative assessment guidelines because of their potential to cause perioperative complications. At the European level, the European Society of Anaesthesiology suggests explicitly asking patients about their use of herbal medicines during pre-operative evaluation, particularly agents that may increase peri-operative bleeding [20]. At the institutional level, this is operationalised in hospital pre-assessment protocols—for example, those of a United Kingdom hospital trust list herbal medicines associated with potential perioperative complications [21].

Moreover, this topic is particularly pertinent in the context of an ageing population. Multimorbidity tends to increase with age and often results in the concurrent use of multiple medications [22], which is itself a major risk factor for drug interactions and adverse events [23]. When combined with FS, herbal products, or specific dietary patterns, this polypharmacy may further increase therapeutic complexity. Several compounds present in FS, particularly medicinal plant extracts, may interfere with cytochrome P450 enzymes, transporters such as P-glycoprotein, or pharmacodynamic mechanisms, thereby altering the efficacy or safety of medicines. Clinically significant food–drug interactions (FDIs) may also occur through mechanisms affecting drug absorption, metabolism, or action [24,25].

HPs play a central role in identifying, preventing, and managing these risks, protecting patient safety, and enhancing the overall quality of healthcare in an era of ubiquitous FS use. However, studies indicate that HPs frequently present knowledge gaps regarding the composition, benefits, and potential interactions of FS, contributing to their undervaluation in clinical practice [26,27,28]. The identification and prevention of potential HDI depend largely on systematic clinical assessment, medication history-taking, and open communication with patients, since their use is not always spontaneously reported [29].

Despite the growing relevance of this topic, the available scientific literature remains fragmented. Published studies have addressed the prevalence of FS use and factors associated with their consumption; however, studies focusing on HPs have often been limited to specific professional groups or specific types of interactions [30,31]. There remains a scarcity of comparative data that integrates multiple health professions and jointly assesses knowledge, attitudes, and practices regarding FDI, HDI, and supplement–drug interactions (SDI); in this study, supplements denote food supplements. In Portugal, published surveys in this area have, to our knowledge, addressed food–drug interactions in restricted populations, namely pharmacy and nutrition professionals [26], nutrition sciences students [27], and registered dietitians [32], and have not extended to herb–drug and supplement–drug interactions or to a professional mix that includes nurses and physicians. This gap is particularly relevant given the increasing use of these products and HPs’ central role in their identification, clinical appraisal, and safe management. Therefore, the primary objective of the present study was to assess the knowledge, attitudes, and clinical practices of Portuguese HPs regarding FDI, HDI, and SDI. As secondary objectives, the study examined whether knowledge differed according to profession, prior specific training in this area, and practice setting.

## 2. Materials and Methods

### 2.1. Sample and Study Design

The present study consisted of a pilot observational, cross-sectional, quantitative investigation aimed at assessing the knowledge, attitudes, and clinical practices of healthcare professionals concerning FDI, HDI, and SDI.

The target population included HPs (physicians, pharmacists, nurses, and nutritionists) actively practising in Portugal, in public, private, or mixed healthcare settings, regardless of their clinical area of practice or prior experience with FS, herbal products, or their interactions with medicines. The inclusion criteria were as follows: (i) active professional practice in Portugal within the specified healthcare professions; (ii) age of 18 years or older; and (iii) provision of informed consent. Incomplete or duplicate responses, as well as those from individuals who did not fit the defined professional profile or who indicated that they were not practising in a health-related field, were excluded. A total of 427 responses were initially recorded. After applying the eligibility criteria, 116 responses were excluded, yielding a final analytical sample of 311 HPs.

Sampling followed a non-probabilistic convenience approach, complemented by snowball sampling. Given the exploratory pilot design and the use of non-probabilistic sampling, no formal a priori sample size calculation was performed. Recruitment remained open throughout the predefined data collection period, and all eligible responses received during that period were included in the analysis.

Participation was voluntary and anonymous, with all responses treated confidentially in accordance with the ethical principles of research. The study was approved by the Ethics Committee for Research of the University of Coimbra (CEIUC), and informed consent was obtained from all participants before data collection.

### 2.2. Data Collection Instruments and Procedures

Data collection took place between 6 November 2025 and 1 March 2026, using a structured, self-administered questionnaire made available online through the LimeSurvey® platform (LimeSurvey Cloud; LimeSurvey GmbH, Hamburg, Germany; https://www.limesurvey.org; version 6.17.14).

The questionnaire was disseminated through digital platforms and via professional associations, healthcare institutions, and professional networks. The platform used enabled the implementation of control mechanisms to minimise duplicate responses.

The instrument was developed specifically for this study and comprised three sections: (1) perceptions, attitudes, and clinical practices; (2) assessment of knowledge regarding FDI, HDI, and SDI (22 items); and (3) sociodemographic and professional characterisation.

The knowledge questions were developed based on the scientific literature on FDI, HDI, and SDI, including reference works on food–, herb–, and supplement–drug interactions [18,33,34], documents from the European Medicines Agency (EMA) [35] and the World Health Organization (WHO) [22], and indexed scientific articles [36,37,38,39,40,41,42,43]. This section of the questionnaire was designed to integrate items on FDI, HDI, and SDI, covering clinically relevant examples from different therapeutic contexts, namely cardiovascular, central nervous system, antibiotic therapy, hormonal therapy, endocrinology/metabolism, and oncology. This approach aimed to assess participants’ knowledge in situations with potential clinical impact across different areas of professional practice and therapeutic contexts, enabling the assessment of knowledge among professionals with potentially distinct educational backgrounds and levels of exposure to the topic. Each knowledge item was assigned to one of the three interaction domains (herb–drug, food–drug, or supplement–drug) according to the nature of the interacting agent as presented in the item stem: items referring explicitly to FS (e.g., garlic, antioxidant, or calcium supplements) were classified as supplement–drug; items referring to foods, beverages, or dietary constituents (e.g., dairy products, coffee, grapefruit, or tyramine-rich foods) as food–drug; and items referring to medicinal plants or herbal preparations designated by their botanical name (e.g., *Hypericum perforatum*, *Valeriana officinalis*) as herb–drug. The 22 knowledge items comprised 11 herb–drug, 8 food–drug, and 3 supplement–drug items. The complete questionnaire is provided in Supplementary File S1.

A pre-test involving 52 HPs was conducted to assess the clarity, comprehensibility, technical functioning, and completion time of the questionnaire. The median completion time was 6.65 min. Qualitative feedback from participants, together with expert content review, led to minor revisions to terminology, botanical nomenclature, and the response options for professional practice setting.

### 2.3. Statistical Analysis

Statistical analyses were performed using IBM SPSS Statistics^®^ (IBM Corp., Armonk, NY, USA; version 26). Categorical variables were summarised as absolute and relative frequencies (*n*, %). The normality of the knowledge score distribution was assessed using the Shapiro–Wilk and Kolmogorov–Smirnov tests; as the distribution deviated significantly from normality, non-parametric methods were adopted, and the score is reported as the median and interquartile range (IQR), with the mean and standard deviation (SD) also provided for descriptive purposes. Between-group comparisons of the knowledge score were performed using the Mann–Whitney *U* test for two-group comparisons and the Kruskal–Wallis *H* test for comparisons across more than two groups; significant Kruskal–Wallis results were further explored using Dunn’s pairwise post hoc test with Bonferroni correction. Differences in performance across the three knowledge domains (FDI, HDI, and SDI) were assessed using the Friedman test on domain scores standardised as percentages of correct responses, with Kendall’s *W* reported as the effect size. Associations between categorical variables were examined using the chi-square (χ^2^) test, with Fisher’s exact test used when expected cell counts fell below five, and Cramér’s *V* reported as the effect size; the relationship between age and knowledge score was evaluated using Spearman’s rank correlation coefficient (ρ). Effect sizes were reported alongside the corresponding significance tests: epsilon-squared (ε^2^) for Kruskal–Wallis comparisons, Spearman’s ρ as a rank-based effect size for two-group comparisons of the knowledge score, and Cramér’s *V* for categorical associations; Kendall’s *W* was reported for the Friedman test, as stated above. For the Spearman ρ effect sizes, 95% confidence intervals were estimated by bias-corrected and accelerated (BCa) bootstrapping with 1000 resamples, whereas confidence intervals were not computed for ε^2^, Cramér’s V, or Kendall’s W. In the analyses of clinical questioning practices, groups were defined by “yes” versus “no” responses, with “do not know” and “not applicable” responses excluded, so that the valid *N* varied across tests and is reported for each. All tests were two-tailed, with statistical significance set at *p* < 0.05.

The level of knowledge was operationalised through a score (knowledge_score), calculated as the sum of correct responses to the 22 items in the knowledge section, ranging from 0 to 22. Correct responses were coded as 1 and incorrect responses as 0. “I do not know” responses were considered incorrect for score calculation, while “not applicable” responses were treated as missing values.

## 3. Results

### 3.1. Sample Characterisation

The final analytical sample comprised 311 HPs. Sociodemographic and professional characteristics are summarised in Table 1. Nurses constituted the largest professional group, and most participants reported no previous academic training or continuing education specifically addressing interactions.

### 3.2. Perceptions, Attitudes, and Clinical Practices of HPs Regarding FDI, HDI, and SDI

Most participants (90.6%) recognised the clinical relevance of HDI and considered that interactions should be addressed in clinical practice. However, routine enquiry was less consistent: 57.6% reported asking patients about the use of FS, multivitamins, or herbal medicines, and 46.0% asked about herbal teas or infusions. Prior experience of suspected interactions, access to sources considered reliable, and previous use of specific resources for checking interactions were reported by fewer than half of the participants. Among those who had used such resources, databases, the Summary of Product Characteristics (SmPC), and patient information leaflets were the most frequently cited. Detailed results are presented in Table 2. Given the clinical relevance of these self-reported practices, an exploratory analysis of their associations with professional and educational variables and with the overall knowledge score was conducted.

### 3.3. Exploratory Analysis of Clinical Questioning Practices

In the exploratory analysis of clinical practices, it was observed that the practice of asking patients about the use of FS, multivitamins, or herbal medicines was significantly associated with profession, access to reliable sources, prior academic training, and continuing education or self-directed learning (*p* < 0.001), but not with the perceived clinical relevance of HDI. Professionals who reported asking about supplements and those who asked about herbal teas or infusions had significantly higher knowledge scores than those who did not (both *p* < 0.001). These associations are detailed in Table 3.

### 3.4. Overall Knowledge Score

The median overall knowledge score was 9.00 (IQR 9.00), with a mean of 9.34 ± 5.91. The score distribution deviated significantly from normality (Shapiro–Wilk, *p* < 0.001), supporting the use of non-parametric methods. Additional descriptive statistics are presented in Table 4.

### 3.5. Internal Consistency of the Knowledge Section

In the final analytical sample, the knowledge section demonstrated high internal consistency following binary recoding of responses as correct or incorrect, with a Cronbach’s alpha coefficient of 0.910 (298 valid cases; listwise deletion).

### 3.6. Determinants of the Knowledge Score

Knowledge scores differed significantly by profession (Kruskal–Wallis *H* = 97.363; *df* = 3; *p* < 0.001; ε^2^ = 0.314). Dunn’s pairwise post hoc tests with Bonferroni correction showed that nurses scored significantly lower than pharmacists, physicians, and nutritionists (all *p* < 0.001), whereas no significant differences were observed among the latter three professions.

Higher knowledge scores were also observed among participants reporting previous academic training specifically addressing interactions (Mann–Whitney *U* = 3137.000; *Z* = −7.737; *p* < 0.001; ρ = 0.439, 95% CI [0.340, 0.523]) and among those reporting continuing education or self-directed learning in this area (U = 3163.000; *Z* = −7.505; *p* < 0.001; ρ = 0.426, 95% CI [0.327, 0.511]). Scores differed across professional practice settings (Kruskal–Wallis *H* = 38.163; *df* = 3; *p* < 0.001; ε^2^ = 0.123), although only the comparison between hospital settings and the heterogeneous “Other” category remained significant after Bonferroni correction (*p* < 0.001).

No significant differences were observed according to length of professional experience (*p* = 0.073) or gender (Mann–Whitney *U* = 6657.500; *Z* = −0.426; *p* = 0.670; ρ = 0.024, 95% CI [−0.104, 0.147]). Age showed a weak negative correlation with the knowledge score (Spearman’s ρ = −0.137; *p* = 0.015). Full descriptive and inferential results are presented in Table A1.

### 3.7. Performance Across Different Knowledge Domains

Performance was compared across the three knowledge domains: food–drug (8 items), herb–drug (11 items), and supplement–drug (3 items) interactions. Because the domains comprised different numbers of items, performance was operationalised as the percentage of correct responses within each domain, placing all three on a common 0–100% scale. The Friedman test indicated statistically significant differences across domains (χ^2^ = 73.512; *df* = 2; *p* < 0.001; *n* = 310; Kendall’s *W* = 0.119), with the highest mean performance in the food–drug domain (49.5% correct), followed by the supplement–drug domain (43.2%) and, lastly, the herb–drug domain (37.7%). The small effect size (Kendall’s *W* = 0.119) indicates that these differences, although statistically significant, were modest in magnitude; given also that the supplement–drug domain comprised only three items, the across-domain comparison—and particularly the relative position of that domain—should be interpreted as exploratory.

### 3.8. Item-by-Item Analysis of the Knowledge Section

Item-level performance varied considerably, with the proportion of correct responses ranging from 21.8% to 72.5%. The highest-performing items concerned valerian and benzodiazepines, coffee and intestinal iron absorption, and dairy products and tetracyclines. Conversely, the lowest-performing items involved chamomile and hormonal therapies, papaya and antihypertensive medicines, green tea and bortezomib, and liquorice and antihypertensive medicines.

No item exceeded 80% correct responses, and ten items had fewer than 40% correct responses. Several of the lowest-performing items were also characterised by high frequencies of “I do not know” responses, particularly those involving green tea and bortezomib, liquorice and antihypertensive medicines, papaya and antihypertensive medicines, grapefruit and statins, and St John’s wort and antiepileptic medicines. Full item-level results are presented in Table 5.

## 4. Discussion

The results of this pilot study show that although most HPs recognise the clinical relevance of FDI, HDI, and SDI, their objective knowledge, as assessed through the technical section, was generally limited. The median knowledge score was 9.00 out of 22 (IQR 9.00), with a mean of 9.34 ± 5.91 provided for descriptive purposes. This result indicates that performance was below half of the maximum possible score and suggests that theoretical appreciation of the problem was not accompanied by equivalent technical knowledge. This finding is particularly relevant because the sample comprised four professional groups—physicians, nurses, pharmacists, and nutritionists—with distinct yet complementary roles in prescribing, dispensing, administering, counselling, and therapeutic monitoring.

These findings are consistent with international literature. In 2023, a systematic review documented persistent gaps in knowledge regarding FDI, HDI, and SDI, alongside heterogeneous beliefs about the consumption of herbs and FS and their interactions with conventional medicines, across different populations and professional settings [44]. Similarly, a recent study involving HPs reported low to moderate levels of knowledge in this area [30]. Two further multiprofessional surveys conducted in 2025 documented limited objective knowledge of FDI coexisting with high recognition of their clinical relevance, alongside explicitly identified educational gaps [45,46]. However, direct comparisons between studies should be made with caution, given the heterogeneity in the instruments used, scoring scales, interaction pairs assessed, and sample composition. The available evidence is, moreover, geographically uneven: studies assessing the knowledge of HPs, and pharmacists in particular, regarding FS and their interactions originate predominantly from Asian and Middle Eastern settings, with comparatively few data from European countries [47].

Direct comparison across these contexts is constrained by differences in undergraduate and postgraduate curricula, regulatory frameworks governing FS and herbal products, and the instruments used to assess knowledge. Despite this heterogeneity, the predominant finding across studies is the presence of persistent knowledge gaps: a survey of community pharmacists in northeastern Italy, for instance, found that only about one-third met the predefined minimum competence standard [48]. A notable exception is a Croatian study in which all participating community pharmacists attained uniformly high knowledge scores, irrespective of professional experience [49], illustrating how markedly performance in this domain may depend on national training and practice contexts. By drawing on a European, multiprofessional sample, the present study adds to an evidence base in which European settings remain comparatively underrepresented.

In the Portuguese context, the findings of this study are particularly consistent with the work of Coelho and Horta, which assessed knowledge of FDIs among pharmacy and nutrition professionals in the Lisbon region, yielding an overall correct response rate of 46.3% [26]. This scenario is reinforced by a more recent national study conducted in 2024: among 151 nutritionists registered with the Portuguese Order of Nutritionists, Beirão et al. reported an average of 58.7% accuracy in specific, clinically relevant FDIs, with markedly lower accuracy for higher-risk interactions, such as propranolol and a high-protein diet (22.5%) and warfarin with garlic or ginger (42.4%). Although 93.4% reported academic exposure to FDIs, only 23.2% considered this training sufficient for practice [32]. Because this single-profession study used a curated, FDI-only instrument, its absolute scores are not directly comparable to the present multiprofessional knowledge score; its value here lies in showing that even a Portuguese professional group with near-universal academic exposure retains specific, clinically meaningful gaps and limited perceived competence. The convergence across these Portuguese studies, obtained using different instruments and samples, suggests that the gaps identified here are unlikely to be artefacts of the present questionnaire. They are more plausibly interpreted as a recurring limitation in integrating knowledge of interactions into the training and practice of HPs in Portugal. This interpretation should nonetheless remain cautious: the available data document relevant knowledge gaps but do not, on their own, support claims of a structural deficiency across the national training system.

One important finding of this study was the discrepancy between perception and practice. Although 90.6% of participants recognised the clinical relevance of herb–drug interactions, only 57.6% reported routinely questioning patients about the use of FS or herbal medicines, and 46.0% about the consumption of teas or infusions. This dissociation suggests that recognising the importance of the problem is not sufficient to ensure its systematic incorporation into clinical practice. Rather than reflecting a lack of awareness of the issue, the results suggest difficulty translating that perception into concrete clinical behaviours, such as screening for exposures, consulting appropriate sources, counselling the patient, and documenting potential risks. This pattern is consistent with international evidence showing that routine inquiry into and documentation of the use of FS and herbal products remain limited, even when their relevance is acknowledged. Stanojević-Ristić et al. reported that only 28% of HPs often or always asked patients on drug therapy about the use of supplements or herbal products, and only 25% recorded this information in the clinical notes [50]. As patient disclosure depends heavily on being asked, this incomplete screening is clinically meaningful, since non-disclosure is frequent when professionals do not actively enquire [14]. Consistent with this, when disclosure has been examined directly, only a minority of herbal-product users—in one study of university students—reported their use to a healthcare provider [51].

The exploratory analysis reinforces this interpretation: questioning patients about FS was not merely associated with regarding interactions as clinically relevant, but rather with professional background, access to reliable sources, and prior or ongoing training; moreover, professionals who routinely questioned patients about FS or teas/infusions scored significantly higher on knowledge. Because these associations are cross-sectional and derived from self-reported practices, their direction cannot be established: greater knowledge may promote more systematic questioning, questioning may in turn reinforce knowledge, and both may be jointly shaped by prior training and professional role. Clinical practice in this domain therefore appears to depend less on a generic perception of the topic’s importance than on applicable knowledge, technical confidence, and informational support. This dissociation between attitude and technical preparedness has also been described in other contexts. A study involving HPs in the United Kingdom in 2019 found that many recognised the relevance of herbal medicines, despite a substantial proportion having received no formal training in this area [31]. Similarly, a 2024 qualitative review by Shen et al. indicated that HPs tend to acknowledge the existence of HDIs, but frequently report a lack of specific knowledge, uncertainty in communicating with patients, and an absence of practical decision-support tools [52]. The findings of the present study thus fit within a broader pattern: awareness of the problem exists, yet limitations in the clinical operationalisation of that knowledge persist.

The differences observed between professional groups warrant particular attention. Knowledge scores differed significantly across professions, with pharmacists achieving the highest scores and nurses the lowest, while physicians and nutritionists occupied an intermediate position; the three higher-performing groups did not differ significantly from one another. This distribution is plausible, given pharmacists’ greater curricular exposure to pharmacology, pharmacokinetics, medication safety, and interactions, as well as the closer proximity of nutritionists to the dietary determinants of therapeutics. International studies have also described superior performance by pharmacists in FDI assessments, as reported by Balaban et al. in a study involving Turkish HPs [30]. A comparable professional gradient was first described in 2000 by Couris et al. in a randomly selected, balanced four-profession US survey of warfarin–vitamin K interaction knowledge, in which pharmacists scored highest and nurses lowest of the groups assessed, whereas dietitians performed best specifically on the food-interaction items [53]. Roughly a quarter of a century later, the nurse-specific picture appears largely unchanged: Subih et al., in a 2025 study of hospital nurses in Jordan, reported only moderate overall knowledge of warfarin interactions, with substantially weaker recognition of warfarin–drug than of warfarin–food interactions, and identified training, formal education, direct clinical exposure, self-confidence, and greater work experience—rather than age, gender, or job position—as the principal determinants of knowledge [54]. The two studies are not directly comparable, differing both in design (a balanced four-profession sample versus a single-profession convenience sample) and in scoring scales; their convergence across 25 years nonetheless reinforces two observations central to the present findings: nurses recur as a lower-performing group in warfarin-related knowledge, and this gap is associated chiefly with modifiable educational factors rather than with fixed professional or demographic attributes [53,54].

Coelho and Horta’s data, however, complicate this picture, with nutritionists outperforming pharmacy professionals in certain FDI domains [26]. This suggests that different professional training backgrounds may confer distinct advantages: pharmacists may be better prepared to identify classical pharmacological interactions, whereas nutritionists may have greater sensitivity to interactions mediated by foods, nutrients, and dietary patterns. Consistent with this, a large survey of physicians, pharmacists, and nurses likewise found that knowledge and practices regarding FS varied markedly across professional groups, underscoring the uneven distribution of competencies in this area [55]. Studies focusing specifically on physicians point in the same direction: in a cross-sectional study of physicians in 2021 in Rasht, Iran, participants held favourable attitudes towards herbal remedies but lacked sufficient knowledge to counsel patients on their use, with no significant differences according to experience, workplace, or field of practice [56]. A multicentre Turkish study of surgical doctors and nurses similarly reported low overall knowledge of nutrients and supplements in a surgical setting yet found that nurses outperformed physicians specifically on SDI items—an advantage the authors linked to nurses’ routine role in medication administration and adverse-effect monitoring—while training, rather than professional category or formal education level, was the factor most consistently associated with higher knowledge [57]. The divergence of these patterns from the present findings, where nurses scored lowest, reinforces that the professional gradient observed here is not universal but is shaped by national training and practice contexts and by the specific competencies each role exercises. Notably, the higher scores on drug-interaction items reported among female doctors and nurses by Büyükkasap and Yazıcı [57] were not reproduced here, as knowledge scores did not differ significantly by gender in the present sample.

The lower performance of nurses should be interpreted with caution. On the one hand, this result may reflect reduced formal curricular exposure to applied pharmacology, phytotherapy, and FDI, HDI, and SDI. On the other hand, the present study had a predominance of professionals working in hospital settings, which may have influenced the types of clinical exposure and contact with situations involving self-medication, FS, and herbal products. Even so, the gap is clinically relevant, given that nurses play a central role in medication administration, adverse effect surveillance, health education, and the detection of clinical changes throughout the care pathway. These findings should not be interpreted as reflecting an inherent limitation of the nursing profession, but rather as identifying a potentially modifiable educational gap. They therefore highlight an opportunity to strengthen competencies within a professional group that has direct, frequent, and sustained contact with patients.

Beyond these professional-group differences, three further exploratory strands of analysis help to characterise the observed knowledge: its distribution across interaction domains, its variation by practice setting, and its association with demographic factors.

The domain-specific analysis further underscores the heterogeneity of the observed knowledge. After standardisation, the highest relative performance was recorded in the FDI domain and the lowest in the HDI domain, with the SDI domain in an intermediate position; this last comparison should be read with caution, as the SDI domain comprised only three items. The robust contrast is therefore between FDI and HDI, suggesting that classical FDI, more frequently taught and discussed, are more readily recognised, whereas HDI remain a less consolidated area. The high frequency of “I do not know” responses, particularly in the phytotherapy items, is especially informative, as it reflects explicitly acknowledged uncertainty rather than mere error and may favour the uptake of targeted educational interventions. This pattern is consistent with the international literature: In 2026, Qudah et al. reported moderate overall knowledge among community pharmacists in the United Arab Emirates but substantial gaps in less recognised interactions, such as that between spironolactone and potassium-rich foods (correctly identified by only 25.9%) [58], indicating that even groups close to pharmacotherapy show gaps when interactions are less frequently taught or protocolised. A similar contrast is seen in Portugal, where classical interactions (e.g., tetracyclines and dairy products) are more readily recognised than others involving central nervous system drugs [26].

In the herb–drug domain, concern is compounded by the widespread social perception that “natural” products are, by definition, safe. This perception may reduce the likelihood that patients will spontaneously disclose the use of medicinal plants, teas, supplements, or other natural products to HPs. Sile et al., in a study involving Latvian citizens, identified high rates of herbal medicinal product use and highlighted the risk of clinically relevant combinations involving, among others, grapefruit, St John’s wort, and valerian [59]. This concern is compounded by the limited preparedness of frontline professionals: a scoping review of community pharmacists conducted in 2025 found that, despite generally positive attitudes and recognition of their counselling role, pharmacists’ knowledge of FS is frequently insufficient—constrained by limited training, scarce nutrition education, and poor access to reliable information sources [47]. These examples are particularly valuable for discussion, as they illustrate that some seemingly innocuous substances may have relevant pharmacokinetic or pharmacodynamic implications. In the context of the present study, the lower performance in the herb–drug domain reinforces the need to afford greater educational and clinical visibility to these interactions.

Differences across professional practice settings should likewise be interpreted with caution. The higher mean scores observed among participants in the “Other” category and in primary healthcare settings, compared to those working in hospital contexts, may reflect differences in exposure to situations involving counselling, self-medication, supplements, and natural products. However, the “Other” category is heterogeneous and does not permit robust inferences about specific professional settings. Furthermore, the cross-sectional design and convenience sampling preclude establishing causal relationships between practice setting and knowledge level. Nevertheless, this finding raises a relevant hypothesis for future research: the care setting may influence how often professionals encounter questions about nutrition, supplementation, phytotherapy, and medication safety.

Age showed only a weak negative correlation with knowledge (ρ = −0.137), accounting for less than 2% of the variance, and length of professional experience was not significantly associated with performance; neither seniority nor age, therefore, emerged as a meaningful determinant of knowledge in this sample. This contrasts with the positive associations between seniority and knowledge reported elsewhere [54,57], and reinforces that, in this sample, competence tracked modifiable academic and continuing education rather than accumulated age or experience.

The association between prior training and better performance represents one of the findings with the greatest practical relevance. Both academic training on interactions and continuing education or self-directed learning were associated with higher knowledge scores. This finding suggests that knowledge in this area is modifiable and may benefit from structured educational interventions. In 2025, Çakır and Memiş demonstrated that targeted training interventions can improve FDI knowledge among pharmacists and pharmacy students [60]. Although the settings and populations are not directly comparable, this type of evidence supports the notion that specific training programmes—oriented towards clinical cases and frequently encountered interaction pairs—may prove valuable in improving professional practice. The scale of the underlying training deficit is illustrated by a survey among pharmacists in 2024, in which just over a quarter reported having received no coverage of FDI during their undergraduate education, and only about one in six had undertaken any formal training on the topic after entering practice—reinforcing that the observed gaps are, to a considerable extent, a remediable consequence of limited prior and continuing exposure [61].

The clinical implications of the findings are clear. The low self-reported use of specific resources and the limited access to sources perceived as reliable highlight the need to strengthen practical decision-support tools accessible to different professional groups. These tools should not be confined to complex or restricted-access databases; wherever possible, they should be integrated into real-world practice settings, such as outpatient consultations, dispensing, medication reconciliation, hospital admission, and discharge education. This need is particularly pertinent for FS, whose labelling remains regulatorily complex and insufficiently harmonised—especially regarding health claims and permitted dosages—making product information difficult for professionals to interpret; in this regard, transparent and accessible information resources, such as databases providing standardised label data, offer a valuable model for supporting accurate professional advice [62]. The guideline on the investigation of drug interactions issued by the EMA Committee for Medicinal Products for Human Use (CHMP) recommends that the potential for drug–drug and drug–food interactions be evaluated during drug development—including for herbal medicinal products—and that the findings be translated into appropriate therapeutic recommendations in the product information including the SmPC [35]. Although this regulatory framework primarily focuses on drug assessment, it underscores the subject’s clinical and institutional relevance.

From an educational standpoint, the results point to the need to strengthen the coverage of FDI, HDI, and SDI in the undergraduate and postgraduate training of HPs. This recommendation should, however, be implemented in a balanced manner: rather than supporting broad curricular reform, the data reinforce the relevance of integrating more applied, cross-cutting, and interprofessional content on interactions—drawing on clinical examples, decision algorithms, and validated reference sources. The goal should not be to turn all professionals into specialists in phytotherapy or clinical pharmacology, but rather to ensure a minimum set of competencies that enables them to identify risks, actively question patients, recognise warning signs, and refer appropriately or consult appropriate sources.

### 4.1. Limitations

This study has limitations that must be considered. It is a pilot, cross-sectional study based on a self-administered questionnaire, which limits causal inference. Convenience and snowball sampling, together with dissemination through digital channels, limit the generalisability of the findings to all HPs in Portugal and may have introduced selection bias, namely an overrepresentation of professionals with greater interest in or awareness of the topic, which could have led to an overestimation of the observed knowledge levels. Because recruitment relied on an openly disseminated online link with snowball propagation, the number of individuals who received the invitation is unknown, and a formal response rate could not be calculated. A predominance of nurses, female participants, and hospital-based professionals was also observed; although this broadly mirrors the structure of the Portuguese National Health Service (SNS) workforce—heavily centred on nurses and markedly feminised, with women accounting for approximately 78% of all professionals [63]—it may have influenced the overall results and constrained the balance between professional groups. In addition, some categories—such as the “Other” practice setting—are heterogeneous and should be interpreted with caution. All associations were examined bivariately; no multivariable or adjusted analyses were performed, so the contributions of profession, practice setting, and training to knowledge cannot be disentangled and may be mutually confounded, and the observed between-profession differences should therefore be regarded as exploratory, particularly given the marked numerical imbalance among the professional groups. Future studies with larger, more balanced samples should employ multivariable models to adjust for the mutual confounding among profession, practice setting, and training. The supplement–drug interaction domain comprised only three items, so the across-domain comparison—and the relative position of that domain in particular—should likewise be treated as exploratory (Section 3.7). The selected interactions also differ in the strength of the evidence that supports them, ranging from well-documented associations to others supported mainly by mechanistic reasoning, preclinical data, or isolated case reports; the answer key was defined on a precautionary basis, assuming clinical vigilance rather than requiring confirmatory trial evidence, so elevated “I do not know” responses to some items may partly reflect legitimate scientific uncertainty rather than a knowledge deficit alone. Moreover, the knowledge instrument, although internally consistent, was developed specifically for this study and has not been externally validated, and the self-reported assessment of attitudes and practices may be subject to social desirability and recall biases. Formal educational level was not recorded as a separate variable; as entry qualifications are profession-specific in Portugal, educational level is largely confounded with profession and could not be examined independently.

### 4.2. Future Perspectives

An additional consideration for future research concerns the quality and compositional reliability of FS and herbal products. Although not all quality defects constitute interactions, discrepancies between labelled and actual composition [64], contamination with microorganisms, mycotoxins, or pesticide residues [65], and the presence of potentially toxic elements [66] may alter the exposure profile and complicate the causal attribution of adverse events. Adulteration with undeclared pharmacologically active substances [67] may additionally generate unexpected drug–drug interactions. The present instrument did not address this dimension of risk; future work should therefore examine whether HPs recognise product quality as an additional determinant of product safety and, where undeclared pharmacologically active substances are present, of interaction risk. Such research could be complemented by continued analytical surveillance of product identity, composition, and chemical and microbiological purity.

## 5. Conclusions

The findings of this study suggest that the HPs surveyed broadly recognise the clinical relevance of FDI, HDI, and SDI, yet there are objective knowledge gaps and an incomplete integration of this topic into clinical practice. The median knowledge score, the discrepancy between perceived clinical relevance and reported practice, and the lower relative performance observed in the herb–drug domain indicate that knowledge and clinical implementation in this area remain uneven among the professionals included in the sample.

The association between prior training, continuing education, or self-directed learning and better performance reinforces the importance of targeted educational strategies adapted to the needs of different professional groups. Likewise, the high frequency of “I do not know” responses across several items indicates explicitly acknowledged uncertainty regarding several interaction pairs, which may represent an opportunity for targeted educational interventions.

This study contributes to the characterisation of an underexplored area in Portugal by adopting a multiprofessional approach to HPs’ knowledge, attitudes, and clinical practices regarding FDI, HDI, and SDI. Within the limits of the sample examined, the findings reinforce the need to promote applied training, improve access to reliable information sources, and support the systematic screening of the use of FS, herbal products, teas, infusions, and other natural products in clinical practice. Building on these exploratory findings, future research should pursue more representative national studies, further validation of the knowledge instrument, and the evaluation of targeted educational interventions to strengthen the safe concomitant use of natural products and conventional medicines.

## Figures and Tables

**Table 1:** Sociodemographic and professional characterisation of the sample (*n* = 311).

	Total Sample (*n* = 311)
Gender, % (*n*)	
Female	82.3 (256)
Male	17.4 (54)
No response	0.3 (1)
Age (years)	43.2 ± 11.0
Profession, % (*n*)	
Nurse	57.2 (178)
Pharmacist	22.5 (70)
Nutritionist	12.5 (39)
Physician	7.7 (24)
Years of professional experience, % (*n*)	
<5	13.8 (43)
5–10	10.3 (32)
11–20	25.7 (80)
21–30	31.5 (98)
>30	18.6 (58)
Professional practice setting, % (*n*)	
Hospital unit	54.3 (169)
Primary healthcare	12.2 (38)
Social solidarity institution	4.2 (13)
Other	29.3 (91)
Academic training on interactions, % (*n*)	
Yes	21.5 (67)
No	78.5 (244)
Continuing education/self-directed learning on interactions, % (*n*)	
Yes	20.9 (65)
No	79.1 (246)

Data are expressed as mean ± SD or % (*n*), as appropriate. The “Other” practice-setting category grouped settings not listed as separate response options; as no free-text specification was collected, its internal composition could not be characterised.

**Table 2:** Perceptions, attitudes, and clinical practices regarding interactions between food supplements, herbal products, and medicines.

	Total Sample (*n* = 311)
HDI are clinically relevant, % (*n*)	
Yes	90.6 (281)
No	2.3 (7)
Do not know	7.1 (22)
Importance of addressing this topic during consultations or in clinical intervention, % (*n*)	
Yes	89.6 (275)
No	1.0 (3)
Do not know	9.4 (29)
Consider this risk to be undervalued by HPs, % (*n*)	
Yes	85.9 (267)
No	7.7 (24)
Do not know	6.4 (20)
Routinely ask patients whether they take FS, multivitamins, or herbal medicines, % (*n*) ^(a)^	
Yes	57.6 (174)
No	42.1 (127)
Do not know	0.3 (1)
Ask patients about their consumption of herbal teas or infusions, % (*n*) ^(a)^	
Yes	46.0 (139)
No	54.0 (163)
Prior experience with potential interactions, % (*n*) ^(b)^	
Interaction between FS/multivitamins/herbal medicines and conventional medicines	38.9 (121)
Interaction between herbal teas/infusions and conventional medicines	34.1 (106)
Access to sources considered reliable for checking interactions, % (*n*)	
With access	34.1 (105)
Without access	46.8 (144)
Do not know	19.2 (59)
Prior use of a specific resource to verify interactions, % (*n*)	
Yes ^(c)^	41.9 (129)
Databases	48.8 (63)
Summary of Product Characteristics (SmPC)	46.5 (60)
Patient information leaflet	41.9 (54)
Pharmacy computer system	38.0 (49)
Books	23.3 (30)
No	55.2 (170)
Do not know	2.9 (9)

Data are expressed as % (*n*) based on valid responses. ^(a)^ “Not applicable” responses were excluded. ^(b)^ Proportion reporting a witnessed or suspected interaction; remaining responses were “no” or “do not know”. ^(c)^ Multiple responses were permitted; percentages may exceed 100%.

**Table 3:** Associations of clinical questioning practices.

Practice/Association	Test Statistic	*p*	Effect Size (95% CI)
Asking about FS, multivitamins, or herbal medicines, by:			
Profession	χ^2^ (3) = 37.087	<0.001	V = 0.351
Access to reliable sources	χ^2^ (2) = 31.390	<0.001	V = 0.324
Prior academic training	χ^2^ (1) = 21.704	<0.001	V = 0.269
Continuing education/self-directed learning	χ^2^ (1) = 30.357	<0.001	V = 0.318
Perceived clinical relevance of HDI	Fisher’s exact	0.218	V = 0.098
Knowledge score, by questioning practice:			
Asking about supplements (yes vs. no)	U = 7183.500; *Z* = −5.191	<0.001	ρ = 0.300 [0.203, 0.398]
Asking about herbal teas/infusions (yes vs. no)	U = 7779.000; *Z* = −4.700	<0.001	ρ = 0.271 [0.153, 0.393]

χ^2^: chi-square test; *df*: degrees of freedom; U: Mann–Whitney statistic; Z: Mann–Whitney *Z* statistic; V: Cramér’s V; ρ: Spearman’s correlation coefficient; HDI: herb–drug interactions. Effect sizes: Cramér’s *V* for chi-square/Fisher associations and Spearman’s ρ for the two-group knowledge-score comparisons; 95% CIs for ρ were obtained by BCa bootstrap (1000 resamples), whereas CIs were not estimated for Cramér’s V. For both comparisons, ρ is reported as a positive magnitude; participants who reported the practice scored higher than those who did not. Valid *N* varies across rows owing to missing data on the respective predictor. The association between asking about supplements and perceived clinical relevance of HDI was tested using Fisher’s exact test owing to expected cell counts <5 (χ^2^ = 2.861; *df* = 2). Mann–Whitney *U* tests compared knowledge scores between professionals who did and those who did not report each questioning practice.

**Table 4:** Descriptive statistics of the overall knowledge score (*n* = 311).

Parameter	Value
Minimum score	0
Maximum score	22
Mean	9.34
Standard deviation	5.91
Median	9.00
Interquartile range	9.00
95% CI for the mean—lower bound	8.68
95% CI for the mean—upper bound	10.00
Kolmogorov–Smirnov	*p* = 0.001
Shapiro–Wilk	*p* < 0.001

95% CI: 95% confidence interval; *p* < 0.05 indicates statistically significant deviation from normality.

**Table 5:** Distribution of correct, incorrect, and “I do not know” responses per item in the knowledge section.

Item	Interaction Assessed	Domain	Valid *n*	Responses, % (*n*)		
				Correct	Incorrect	“I Do Not Know”
1	*Hypericum perforatum* L. and anxiolytic medicines	Herb–drug	310	32.6 (101)	22.6 (70)	44.8 (139)
2	*Citrus × paradisi* Macfad. and cardiovascular medicines	Food–drug	310	46.1 (143)	10.6 (33)	43.2 (134)
3	*Camellia sinensis* and warfarin	Herb–drug	309	32.4 (100)	29.8 (92)	37.9 (117)
4	Dairy products and tetracyclines	Food–drug	310	66.1 (205)	6.1 (19)	27.7 (86)
5	Coffee and intestinal iron absorption	Food–drug	308	66.9 (206)	8.8 (27)	24.4 (75)
6	*Matricaria recutita* and hormonal therapies	Herb–drug	307	21.8 (67)	22.5 (69)	55.7 (171)
7	*Allium sativum* and warfarin	Supplement–drug	310	48.1 (149)	14.2 (44)	37.7 (117)
8	*Ginkgo biloba* and SSRIs	Herb–drug	310	39.0 (121)	11.6 (36)	49.4 (153)
9	*Glycyrrhiza glabra* L. and antihypertensive medicines	Herb–drug	308	28.9 (89)	3.2 (10)	67.9 (209)
10	*Cinnamomum* spp. and oral antidiabetics	Food–drug	309	47.2 (146)	14.9 (46)	37.9 (117)
11	*Valeriana officinalis* L. and benzodiazepines	Herb–drug	309	72.5 (224)	7.4 (23)	20.1 (62)
12	*Camellia sinensis* and bortezomib	Herb–drug	310	25.5 (79)	5.8 (18)	68.7 (213)
13	Antioxidant supplements and chemotherapy	Supplement–drug	310	49.7 (154)	7.1 (22)	43.2 (134)
14	*Ginkgo biloba* and ibuprofen	Herb–drug	310	44.5 (138)	5.8 (18)	49.7 (154)
15	Tyramine-rich foods and MAOIs	Food–drug	310	49.0 (152)	1.0 (3)	50.0 (155)
16	*Citrus × paradisi* Macfad. and statins	Food–drug	309	35.3 (109)	6.8 (21)	57.9 (179)
17	Calcium supplements and levothyroxine	Supplement–drug	307	31.9 (98)	14.0 (43)	54.1 (166)
18	Milk/dairy products and quinolone/tetracycline absorption	Food–drug	310	59.0 (183)	3.9 (12)	37.1 (115)
19	*Hypericum perforatum* L. and antiepileptics	Herb–drug	310	40.3 (125)	2.6 (8)	57.1 (177)
20	*Matricaria recutita* and benzodiazepines	Herb–drug	310	43.5 (135)	12.9 (40)	43.5 (135)
21	*Carica papaya* and antihypertensive medicines	Food–drug	309	24.9 (77)	12.0 (37)	63.1 (195)
22	*Senna alexandrina* and oral corticosteroid absorption/efficacy	Herb–drug	308	34.1 (105)	10.1 (31)	55.8 (172)

Data are expressed as % (*n*), calculated on the valid *n* for each item; for each item, the correct, incorrect, and “I do not know” counts sum to the valid *n*; owing to rounding, the corresponding percentages may not total exactly 100%. Missing responses correspond to items not answered or marked "not applicable". Abbreviations: MAOIs, monoamine oxidase inhibitors; SSRIs, selective serotonin reuptake inhibitors.

## Data Availability

The data presented in this study are not publicly available due to privacy and ethical restrictions, as they contain individual-level survey responses from HPs. Aggregated data supporting the findings of this study are available from the corresponding author upon reasonable request.

## Supplementary Materials

The following supporting information can be downloaded at: https://www.mdpi.com/article/10.3390/nu18162600/s1, File S1: Questionnaire.

## Abbreviations

The following abbreviations are used in this manuscript:

95% CI	95% Confidence Interval
BCa	Bias-corrected and accelerated bootstrap
CEIUC	Ethics Committee for Research of the University of Coimbra
CHMP	Committee for Medicinal Products for Human Use
*df*	Degrees of freedom
EMA	European Medicines Agency
EU	European Union
FDI	Food–Drug Interaction
FS	Food Supplements
*H*	Kruskal–Wallis statistic
HDI	Herb–Drug Interaction
HPs	Healthcare Professionals
IAN-AF	National Food and Physical Activity Survey (Inquérito Alimentar Nacional e de Atividade Física)
IQR	Interquartile Range
MAOIs	Monoamine Oxidase Inhibitor
SD	Standard Deviation
SDI	Supplement–Drug Interaction
SmPC	Summary of Product Characteristics
SNS	Portuguese National Health Service
SSRIs	Selective Serotonin Reuptake Inhibitor
*U*	Mann–Whitney statistic
US	United States
*V*	Cramér’s *V* (effect size)
*W*	Kendall’s *W* (effect size)
WHO	World Health Organisation
*Z*	Mann–Whitney *Z* statistic
ε^2^	Epsilon-squared (effect size)
ρ	Spearman’s correlation coefficient
χ^2^	Chi-square statistic

## Appendix A

**Table A1:** Comparison of knowledge scores according to professional, educational and sociodemographic variables.

Variable	Category	*N*	Mean ± *SD*	Median (*IQR*)	Statistical Test	*p*	Effect Size (95% CI)
Profession	Nurse	178	6.59 ± 5.15	5.50 (7.25)	H = 97.363; *df* = 3	<0.001	ε^2^ = 0.314
	Pharmacist	70	13.77 ± 4.85	14.00 (7.25)			
	Nutritionist	39	12.56 ± 4.81	11.00 (7.00)			
	Physician	24	11.63 ± 3.99	11.50 (6.50)			
Academic training on interactions	Yes	67	14.28 ± 4.75	14.00 (7.00)	U = 3137.000; *Z* = −7.737	<0.001	ρ = 0.439 [0.340, 0.523]
	No	244	7.99 ± 5.46	8.00 (7.00)			
Continuing education/self-directed learning on interactions	Yes	65	14.34 ± 5.05	15.00 (7.00)	U = 3163.000; *Z* = −7.505	<0.001	ρ = 0.426 [0.327, 0.511]
	No	246	8.02 ± 5.40	8.00 (7.00)			
Professional practice setting	Hospital unit	169	7.65 ± 5.49	7.00 (8.00)	H = 38.163; *df* = 3	<0.001	ε^2^ = 0.123
	Primary healthcare	38	10.03 ± 5.63	11.00 (7.25)			
	Social solidarity institution	13	8.92 ± 6.73	8.00 (10.50)			
	Other	91	12.26 ± 5.54	13.00 (8.00)			
Professional experience (years)	<5	43	10.77 ± 5.73	11.00 (8.00)	H = 8.562; *df* = 4	0.073	ε^2^ = 0.028
	5–10	32	9.44 ± 5.70	10.50 (8.00)			
	11–20	80	9.80 ± 5.70	9.50 (9.00)			
	21–30	98	9.30 ± 6.12	9.00 (10.00)			
	>30	58	7.69 ± 5.89	7.50 (7.25)			
Gender	Female	256	9.44 ± 5.97	9.00 (9.00)	U = 6657.500; *Z* = −0.426	0.670	ρ = 0.024 [−0.104, 0.147]
	Male	54	8.96 ± 5.69	9.00 (7.50)			
Age	—	311	—	—	ρ = −0.137	0.015	ρ = −0.137 [−0.241, −0.029]

Note. SD: standard deviation; IQR: interquartile range; H: Kruskal–Wallis statistic; U: Mann–Whitney statistic; Z: standardised test statistic; *df*: degrees of freedom; ε^2^: epsilon-squared (effect size for Kruskal–Wallis); ρ: Spearman’s correlation coefficient. Dunn–Bonferroni: nurses < all other professions, *p* < 0.001; hospital < Other, *p* < 0.001. Effect sizes are reported as ε^2^ for Kruskal–Wallis comparisons and Spearman’s ρ for two-group and continuous comparisons; 95% CIs for ρ were obtained by bias-corrected and accelerated (BCa) bootstrap (1000 resamples), whereas CIs were not estimated for ε^2^. For the dichotomous predictors, ρ is oriented so that a positive value indicates higher knowledge in the category possessing the characteristic; the direction is corroborated by the group means and medians. For age, the sign of ρ reflects the direction of the correlation (lower scores with increasing age).
