# Supplementary material for: Knowledge, Attitudes, and Clinical Practices Regarding Food–Drug, Herb–Drug, and Supplement–Drug Interactions: A Pilot Cross-Sectional Study Among Healthcare Professionals in Portugal
Source: Nutrients. 2026 Aug 8;18(16):2600. doi: 10.3390/nu18162600 (PMC13516228; doi:10.3390/nu18162600)
Supplement: Supplementary file 1 [file nutrients-18-02600-s001.zip › nutrients-4445666-supplementary.pdf]

Supplementary File S1: Questionnaire

Part A. English Translation of the Questionnaire

Note: This English translation is provided for readers' convenience. The questionnaire was administered to participants in Portuguese. The English version was not used for data collection and has not been separately validated. The substantive wording, response options, question order, and scoring have been preserved.

Project: "Knowledge, Attitudes, and Clinical Practices Regarding Food–Drug, Herb–Drug, and Supplement–Drug Interactions: A Pilot Cross-Sectional Study Among Healthcare Professionals in Portugal".

Principal investigator: Professor Angelina Lopes Simões Pena, PhD

Institution: Faculty of Pharmacy, University of Coimbra

Contact: apena@ff.uc.pt or mcampos@ff.uc.pt

Informed Consent Form

This questionnaire forms part of a study conducted by Walter Matias Silva, a student on the Master's in Herbal Medicinal Products and Plant-Based Food Supplements at the Faculty of Pharmacy of the University of Coimbra, under the supervision of Maria João Campos, MSc, and Professor Angelina Pena, PhD. The study aims to assess the knowledge, attitudes and perceptions of healthcare professionals regarding interactions between foods, herbs, food supplements and medicines — an area of growing relevance to safe clinical practice and to the promotion of integrated healthcare. Participation in this study is restricted to healthcare professionals practising in Portugal (doctors, pharmacists, nurses and nutritionists) and consists of completing an online, closed-response questionnaire lasting approximately 5 minutes. The questionnaire includes questions on professional profile, perceptions and clinical practice, and technical knowledge of interactions. There are no physical, psychological, social or legal risks or discomforts associated with participation. However, should any participant feel uncomfortable with a particular question, they may withdraw at any time, without having to provide a reason. As an immediate benefit, participants may gain knowledge about interactions between foods/herbs/food supplements and conventional medicines. The study complies with the General Data Protection Regulation (GDPR). The questionnaire is anonymous and the data collected will be processed in aggregate form, ensuring that participants cannot be identified. The data will be

used exclusively for academic purposes, within the scope of the master's dissertation. Participation is voluntary and may be discontinued at any time without any penalty. This study was submitted for review to the Ethics Committee for Research of the University of Coimbra (CEIUC), within the scope of its remit, and received a favourable opinion. Should participants wish to access the study results, or have any other questions, they may request these by email: apena@ff.uc.pt (Professor Angelina Pena, PhD) or mcampos@ff.uc.pt (Maria João Campos, MSc). By proceeding, you declare that you understand the information presented above and agree to take part in this study.

Section 1 — General Perceptions and Clinical Practice

Please indicate your response to each question.

1. Do you consider interactions between herbs and medicines to be clinically relevant?
Response options: Yes; No; I do not know; Not applicable.

2. Do you routinely ask patients whether they take food supplements, multivitamins, or herbal medicines?
Response options: Yes; No; I do not know; Not applicable.

3. Do you routinely ask patients whether they consume teas or infusions?
Response options: Yes; No; I do not know; Not applicable.

4. Have you ever observed or suspected an interaction between food supplements, multivitamins, or herbal medicines and conventional medicines?
Response options: Yes; No; I do not know; Not applicable.

5. Have you ever observed or suspected an interaction between teas or infusions and conventional medicines?
Response options: Yes; No; I do not know; Not applicable.

6. Do you consider it important to address this topic during consultations or as part of your clinical intervention?
Response options: Yes; No; I do not know; Not applicable.

7. Do you have access to sources that you consider reliable for checking interactions?
Response options: Yes; No; I do not know; Not applicable.

8. Have you ever used a specific resource to check interactions?
Response options: Yes; No; I do not know; Not applicable.

9. If you answered "Yes" to the previous question, which resource(s) did you use?
Format: Multiple-response question; displayed only when the response to Question 8 was "Yes".
Response options: Patient information leaflet; Summary of Product Characteristics (SmPC); Books; Databases; Pharmacy computer system.

10. Do you consider this risk to be undervalued by healthcare professionals?
Response options: Yes; No; I do not know.

Section 2 — Technical Questions

This section aims to understand healthcare professionals' familiarity with certain technical concepts related to food supplements and medicines.

This is not a knowledge test, and there are no "right" or "wrong" answers in the academic sense. We are interested in how each participant responds to the questions, in order to understand how the information is perceived in professional practice.

Please respond spontaneously and honestly, without consulting searches or supporting materials, as you would in a real work situation.

Thank you very much for your collaboration.

1. St John's wort (*Hypericum perforatum*) enhances the effectiveness of anxiolytic medicines, improving their therapeutic effect.

Response options: True; False; I do not know; Not applicable.

2. Grapefruit (*Citrus* × *paradisi* Macfad.) may enhance the effect of certain cardiovascular medicines.

Response options: True; False; I do not know; Not applicable.

3. Green tea (*Camellia sinensis*) may enhance the effect of oral anticoagulants, such as warfarin, making them more effective.

Response options: True; False; I do not know; Not applicable.

4. Dairy products may reduce the absorption of certain antibiotics, such as tetracyclines.

Response options: True; False; I do not know; Not applicable.

5. Taking iron and coffee at the same time may impair intestinal iron absorption.

Response options: True; False; I do not know; Not applicable.

6. Regular consumption of chamomile (*Matricaria recutita*) may interfere with the effectiveness of hormonal medicines, such as contraceptives or hormone replacement therapy.

Response options: True; False; I do not know; Not applicable.

7. The use of garlic supplements (*Allium sativum*) may increase the risk of bleeding in patients receiving warfarin therapy.

Response options: True; False; I do not know; Not applicable.

8. *Ginkgo biloba* is safe when used with selective serotonin reuptake inhibitors (SSRIs) and does not increase the risk of bleeding.

Response options: True; False; I do not know; Not applicable.

9. Regular consumption of liquorice (*Glycyrrhiza glabra*) may interfere with the effectiveness of antihypertensive medicines.

Response options: True; False; I do not know; Not applicable.

10. Cinnamon (*Cinnamomum* spp.) does not interfere with the action of oral antidiabetic medicines, even when consumed in large quantities.

Response options: True; False; I do not know; Not applicable.

11. The use of valerian (*Valeriana officinalis*) may enhance the sedative effect of medicines such as benzodiazepines.

Response options: True; False; I do not know; Not applicable.

12. Green tea (*Camellia sinensis*) may reduce the effectiveness of certain medicines, such as bortezomib, used in chemotherapy.

Response options: True; False; I do not know; Not applicable.

13. Antioxidant supplements never interfere with cancer treatments such as chemotherapy.

Response options: True; False; I do not know; Not applicable.

14. The use of *Ginkgo biloba* in combination with ibuprofen may increase the risk of bleeding.

Response options: True; False; I do not know; Not applicable.

15. Tyramine-rich foods (such as aged cheeses, cured meats, wine, beer, or soy sauce) should be avoided by patients taking monoamine oxidase inhibitors (MAOIs), as they may cause hypertensive crises.

Response options: True; False; I do not know; Not applicable.

16. Grapefruit (*Citrus* × *paradisi* Macfad.) does not affect the plasma concentration of medicines such as statins.

Response options: True; False; I do not know; Not applicable.

17. Calcium supplements do not interfere with the effectiveness of levothyroxine at low doses.

Response options: True; False; I do not know; Not applicable.

18. The consumption of milk or dairy products improves the absorption of antibiotics such as quinolones and tetracyclines.

Response options: True; False; I do not know; Not applicable.

19. St John's wort (*Hypericum perforatum*) may reduce the effectiveness of antiepileptic medicines such as carbamazepine or phenytoin.

Response options: True; False; I do not know; Not applicable.

20. Combining chamomile (*Matricaria recutita*) with benzodiazepines does not affect the sedative effect on the central nervous system.

Response options: True; False; I do not know; Not applicable.

21. Regular consumption of papaya (*Carica papaya*) may enhance the hypotensive effect of medicines used to treat hypertension.

Response options: True; False; I do not know; Not applicable.

22. Prolonged use of senna (*Senna alexandrina*) may reduce the effectiveness of certain medicines but does not interfere with the absorption of oral corticosteroids.

Response options: True; False; I do not know; Not applicable.

23. Have you ever reported an interaction between food supplements, multivitamins, or herbal medicines and conventional medicines to INFARMED (the Portuguese National Authority of Medicines and Health Products)?

Response options: Yes; No; Not applicable.

24. Have you ever reported an interaction between teas or infusions and conventional medicines to INFARMED (the Portuguese National Authority of Medicines and Health Products)?

Response options: Yes; No; Not applicable.

Section 3 — Sociodemographic Data

1. Profession

Response options: Nurse; Pharmacist; Physician; Nutritionist.

2. Age

Whole-number response, between 20 and 70 years.

3. Gender

Response options: Male; Female; Other; Prefer not to answer.

4. Length of professional experience (years)

Response options: <5; 5–10; 11–20; 21–30; >30.

5. Professional practice setting

Response options: Hospital unit; Primary healthcare (C.S.P.); Private institution of social solidarity (I.P.S.S.); Other.

6. Have you received academic training on interactions between medicines, foods/supplements, and herbs?

Response options: Yes; No.

7. Have you undertaken continuing education or self-directed learning on interactions between medicines, foods/supplements, and herbs?

Response options: Yes; No.

Part B. Original Portuguese Version of the Questionnaire

Complete questionnaire administered to participants.

*The questionnaire was administered in Portuguese and is reproduced below in the language of administration. Minor typographical corrections and standardisation of botanical nomenclature were made for publication; the substantive wording, response options, question order, and scoring were not altered. The questionnaire comprised 41 questions: 10 in Section 1, 24 in Section 2 (including 22 knowledge items and two reporting questions), and seven in Section 3***.**

Termo de Consentimento Informado

Projeto: "Conhecimento, atitudes e práticas clínicas relativamente às interações alimento–medicamento, planta–medicamento e suplemento–medicamento: estudo-piloto transversal entre profissionais de saúde em Portugal".

Investigador Responsável: Profª Doutora Angelina Lopes Simões Pena

Instituição: Faculdade de Farmácia da Universidade de Coimbra

Contacto: apena@ff.uc.pt ou mcampos@ff.uc.pt

O presente questionário faz parte de um estudo realizado pelo estudante Walter Matias Silva, aluno do Mestrado em Medicamentos e Suplementos Alimentares à Base de Plantas da Faculdade de Farmácia da Universidade de Coimbra, sob a orientação da Mestre Maria João Campos e Professora Doutora Angelina Pena. Este estudo tem como objetivo avaliar o conhecimento, atitudes e a perceção dos profissionais de saúde sobre as interações entre alimentos, suplementos alimentares, plantas medicinais e medicamentos, uma área de crescente relevância para a prática clínica segura e para a promoção de cuidados de saúde integrados. A participação neste estudo destina-se exclusivamente a profissionais de saúde em exercício em Portugal (médicos, farmacêuticos, enfermeiros e nutricionistas) e consiste no preenchimento de um questionário online, de resposta fechada, com duração aproximada de 5 minutos. O questionário inclui

perguntas sobre perfil profissional, perceção e prática clínica, e conhecimentos técnicos sobre interações. Não existem riscos ou desconfortos físicos, psicológicos, sociais ou legais associados à participação. No entanto, caso algum participante se sinta desconfortável com alguma questão, poderá interromper a sua participação a qualquer momento, sem necessidade de justificação. Como benefício imediato, os participantes poderão adquirir conhecimento sobre interações entre alimentos/plantas/suplementos e medicamentos convencionais. O estudo cumpre o Regulamento Geral sobre a Proteção de Dados (RGPD). O questionário é anónimo e os dados recolhidos serão tratados de forma agregada, garantindo que não seja possível a identificação dos participantes. Os dados serão utilizados exclusivamente para fins académicos, no âmbito da dissertação de mestrado. A participação é voluntária, podendo ser interrompida a qualquer momento, sem qualquer prejuízo. Este estudo foi submetido a consulta pela Comissão de Ética para a Investigação da Universidade de Coimbra (CEIUC), no âmbito das suas competências, tendo obtido parecer favorável. Caso o participante deseje aceder aos resultados do estudo, assim como outras dúvidas que considerar, poderá solicitá-las através do e-mail: apena@ff.uc.pt (Professora Doutora Angelina Pena) ou mcampos@ff.uc.pt (Mestre Maria João Campos). Ao prosseguir, declara que compreende a informação acima apresentada e aceita participar neste estudo.

Secção 1 — Perceção Geral e Prática Clínica

Indique, por favor, a sua resposta para cada afirmação.

1. Considera que as interações entre plantas e medicamentos são clinicamente relevantes?
Opções de resposta: Sim; Não; Não sei; Não se aplica.

2. Costuma perguntar aos doentes se tomam suplementos alimentares, multivitamínicos ou medicamentos à base de plantas?
Opções de resposta: Sim; Não; Não sei; Não se aplica.

3. Costuma perguntar aos doentes se tomam chás ou infusões?
Opções de resposta: Sim; Não; Não sei; Não se aplica.

4. Já presenciou ou suspeitou de uma interação entre suplementos alimentares, multivitamínicos ou medicamentos à base de plantas e medicamentos?
Opções de resposta: Sim; Não; Não sei; Não se aplica.

5. Já presenciou ou suspeitou de uma interação entre chás ou infusões e medicamentos?
Opções de resposta: Sim; Não; Não sei; Não se aplica.

6. Considera importante abordar este tema nas consultas ou na sua intervenção clínica?
Opções de resposta: Sim; Não; Não sei; Não se aplica.

7. Tem acesso a fontes que considera fidedignas para consultar interações?
Opções de resposta: Sim; Não; Não sei; Não se aplica.

8. Já recorreu a algum recurso específico para verificar interações?
Opções de resposta: Sim; Não; Não sei; Não se aplica.

9. Se respondeu "Sim" à pergunta anterior, quais?

Pergunta de resposta múltipla; aplicável apenas se a resposta à pergunta 8 for "Sim".

Opções de resposta: Folheto informativo; Resumo das Características do Medicamento (RCM); Livros; Bases de dados; Sistema informático da farmácia.

10. Considera que existe subvalorização deste risco por parte dos profissionais?

Opções de resposta: Sim; Não; Não sei.

Secção 2 — Questões Técnicas

Esta secção tem como objetivo compreender o nível de familiaridade dos profissionais com determinados conceitos técnicos relacionados com suplementos alimentares e medicamentos.

Não se trata de uma avaliação de conhecimentos, nem há respostas "certas" ou "erradas" no sentido académico. O que nos interessa é a forma como cada participante reage às perguntas, para compreender como a informação é percebida na prática profissional.

Pedimos apenas que responda de forma espontânea e fiel, sem recorrer a pesquisas ou materiais de apoio, tal como faria numa situação real de trabalho.

Muito obrigado pela sua colaboração!

1. O hipericão (*Hypericum perforatum)* potencia a eficácia dos medicamentos ansiolíticos, melhorando o seu efeito terapêutico.

Opções de resposta: Verdadeiro; Falso; Não sei; Não se aplica.

2. A toranja (*Citrus × paradisi* Macfad.) pode potenciar o efeito de certos medicamentos cardiovasculares.

Opções de resposta: Verdadeiro; Falso; Não sei; Não se aplica.

3. O chá verde (*Camellia sinensis*) pode potenciar o efeito dos anticoagulantes orais, como a varfarina, tornando-os mais eficazes.

Opções de resposta: Verdadeiro; Falso; Não sei; Não se aplica.

4. Os lacticínios podem reduzir a absorção de alguns antibióticos, como as tetraciclinas.

Opções de resposta: Verdadeiro; Falso; Não sei; Não se aplica.

5. A toma simultânea de ferro e café pode comprometer a absorção intestinal do ferro.

Opções de resposta: Verdadeiro; Falso; Não sei; Não se aplica.

6. O consumo regular de camomila (*Matricaria recutita*) pode interferir com a eficácia de medicamentos hormonais, como contracetivos ou terapêutica de reposição hormonal.

Opções de resposta: Verdadeiro; Falso; Não sei; Não se aplica.

7. A administração de suplementos de alho (*Allium sativum*) pode aumentar o risco de hemorragia em doentes sob terapêutica com varfarina.

Opções de resposta: Verdadeiro; Falso; Não sei; Não se aplica.

8. O *Ginkgo biloba* é seguro quando usado com antidepressivos ISRS e não aumenta o risco de hemorragia.

Opções de resposta: Verdadeiro; Falso; Não sei; Não se aplica.

9. O consumo regular de alcaçuz (*Glycyrrhiza glabra*) pode interferir com a eficácia de anti-hipertensores.

Opções de resposta: Verdadeiro; Falso; Não sei; Não se aplica.

10. A canela (*Cinnamomum* spp.) não interfere com a ação dos medicamentos antidiabéticos orais, mesmo quando consumida em grandes quantidades.

Opções de resposta: Verdadeiro; Falso; Não sei; Não se aplica.

11. O uso de valeriana (*Valeriana officinalis*) pode potenciar o efeito sedativo de medicamentos como as benzodiazepinas.
Opções de resposta: Verdadeiro; Falso; Não sei; Não se aplica.

12. O chá verde (*Camellia sinensis*) pode inibir a eficácia de certos fármacos, como o bortezomib, usado em quimioterapia.
Opções de resposta: Verdadeiro; Falso; Não sei; Não se aplica.

13. Os suplementos antioxidantes nunca interferem com tratamentos oncológicos, como a quimioterapia.
Opções de resposta: Verdadeiro; Falso; Não sei; Não se aplica.

14. A administração de *Ginkgo biloba* em associação com ibuprofeno pode aumentar o risco de hemorragia.
Opções de resposta: Verdadeiro; Falso; Não sei; Não se aplica.

15. A ingestão de alimentos ricos em tiramina (como queijos maturados, enchidos, vinho, cerveja ou molho de soja) deve ser evitada em doentes sob IMAO, pois pode causar crises hipertensivas.
Opções de resposta: Verdadeiro; Falso; Não sei; Não se aplica.

16. A toranja (*Citrus × paradisi* Macfad.) não afeta a concentração plasmática de medicamentos como as estatinas.
Opções de resposta: Verdadeiro; Falso; Não sei; Não se aplica.

17. A toma de suplementos de cálcio não interfere com a eficácia da levotiroxina em doses baixas.
Opções de resposta: Verdadeiro; Falso; Não sei; Não se aplica.

18. A ingestão de leite ou derivados melhora a absorção de antibióticos como quinolonas e tetraciclinas.
Opções de resposta: Verdadeiro; Falso; Não sei; Não se aplica.

19. O hipericão (*Hypericum perforatum*) pode reduzir a eficácia de antiepilépticos como a carbamazepina ou a fenitoína.
Opções de resposta: Verdadeiro; Falso; Não sei; Não se aplica.

20. A combinação de camomila (*Matricaria recutita*) com benzodiazepinas não interfere com o efeito sedativo no sistema nervoso central.
Opções de resposta: Verdadeiro; Falso; Não sei; Não se aplica.

21. A ingestão regular de papaia (*Carica papaya*) pode potenciar o efeito hipotensor de medicamentos usados no tratamento da hipertensão arterial.
Opções de resposta: Verdadeiro; Falso; Não sei; Não se aplica.

22. O uso prolongado de sene (*Senna alexandrina)* pode reduzir a eficácia de alguns medicamentos, mas não interfere com a absorção de corticosteroides orais.
Opções de resposta: Verdadeiro; Falso; Não sei; Não se aplica.

23. Alguma vez reportou uma interação entre suplementos alimentares, multivitamínicos ou medicamentos à base de plantas e medicamentos ao INFARMED?
Opções de resposta: Sim; Não; Não se aplica.

24. Alguma vez reportou uma interação entre chás ou infusões e medicamentos ao INFARMED?
Opções de resposta: Sim; Não; Não se aplica.

Secção 3 — Dados Sociodemográficos

1. Profissão

Opções de resposta: Enfermeiro; Farmacêutico; Médico; Nutricionista.

2. Idade

Resposta numérica inteira, entre 20 e 70 anos.

3. Género

Opções de resposta: Masculino; Feminino; Outro; Prefiro não responder.

4. Tempo de experiência profissional (em anos)

Opções de resposta: <5; 5–10; 11–20; 21–30; >30.

5. Local de exercício profissional

Opções de resposta: Unidade hospitalar; C.S.P.; I.P.S.S.; Outro.

6. Já teve formação académica sobre interações entre medicamentos, alimentos/suplementos e plantas?

Opções de resposta: Sim; Não.

7. Já teve formação contínua ou autoaprendizagem sobre interações entre medicamentos, alimentos/suplementos e plantas?

Opções de resposta: Sim; Não.
